# Cholesterol sulfate limits neutrophil recruitment and gut inflammation during mucosal injury

**DOI:** 10.3389/fimmu.2023.1131146

**Published:** 2023-03-17

**Authors:** Kenji Morino, Kazufumi Kunimura, Yuki Sugiura, Yoshihiro Izumi, Keisuke Matsubara, Sayaka Akiyoshi, Rae Maeda, Kenichiro Hirotani, Daiji Sakata, Seiya Mizuno, Satoru Takahashi, Takeshi Bamba, Takehito Uruno, Yoshinori Fukui

**Affiliations:** ^1^ Division of Immunogenetics, Department of Immunobiology and Neuroscience, Medical Institute of Bioregulation, Kyushu University, Fukuoka, Japan; ^2^ Multiomics Platform, Center for Cancer Immunotherapy and Immunobiology, Graduate School of Medicine, Kyoto University, Kyoto, Japan; ^3^ Division of Metabolomics, Research Center for Transomics Medicine, Medical Institute of Bioregulation, Kyushu University, Fukuoka, Japan; ^4^ Laboratory Animal Resource Center in Transborder Medical Research Center, Institute of Medicine, University of Tsukuba, Tsukuba, Japan

**Keywords:** cholesterol sulfate, SULT2B1, DOCK2, neutrophil, gut inflammation, mass spectrometry, CyTOF

## Abstract

During mucosal injury, intestinal immune cells play a crucial role in eliminating invading bacteria. However, as the excessive accumulation of immune cells promotes inflammation and delays tissue repair, it is essential to identify the mechanism that limits the infiltration of immune cells to the mucosal-luminal interface. Cholesterol sulfate (CS) is the lipid product of the sulfotransferase SULT2B1 and suppresses immune reactions by inhibiting DOCK2-mediated Rac activation. In this study, we aimed to elucidate the physiological role of CS in the intestinal tract. We found that, in the small intestine and colon, CS is predominantly produced in the epithelial cells close to the lumen. While dextran sodium sulfate (DSS)-induced colitis was exacerbated in *Sult2b1*-deficient mice with increased prevalence of neutrophils, the elimination of either neutrophils or intestinal bacteria in *Sult2b1*-deficient mice attenuated disease development. Similar results were obtained when the *Dock2* was genetically deleted in *Sult2b1*-deficient mice. In addition, we also show that indomethacin-induced ulcer formation in the small intestine was exacerbated in *Sult2b1*-deficient mice and was ameliorated by CS administration. Thus, our results uncover that CS acts on inflammatory neutrophils, and prevents excessive gut inflammation by inhibiting the Rac activator DOCK2. The administration of CS may be a novel therapeutic strategy for inflammatory bowel disease and non-steroidal anti-inflammatory drug-induced ulcers.

## Introduction

The mammalian intestine harbors approximately 10^14^ commensal bacteria (1). To prevent ‘unwanted’ immune responses, these bacteria are spatially segregated in the intestinal lumens through several types of barriers, including tight junctions, mucins, antimicrobial peptides, and flagellar-binding proteins (2–5). However, upon mucosal injury, these bacteria readily invade into the intestinal tissues. Although immune cells, such as neutrophils, play crucial roles in eliminating these bacteria, excessive immune cell accumulation promotes inflammation and delays tissue repair (6), which results in inflammatory bowel diseases (IBD), such as ulcerative colitis (UC) and Crohn’s disease (7, 8). Additionally, excessive neutrophil infiltration into ulcerative lesions in the small intestine (SI) aggravates non-steroidal anti-inflammatory drug (NSAID)-induced ulcers (9, 10). Therefore, the mechanism limiting immune cell infiltration in the mucosal-luminal interface needs to be identified.

Dedicator of cytokinesis protein 2 (DOCK2) is a Rac-specific guanine nucleotide exchange factor (GEF) and is predominantly expressed in hematopoietic cells (11, 12). Although DOCK2 does not contain the Dbl homology domain typically observed in GEFs, DOCK2 mediates the GTP-GDP exchange reaction for Rac *via* its DOCK homology region (DHR)-2 domain (12, 13). Activated Rac regulates various cellular functions by remodeling the actin cytoskeleton (14, 15). Various studies indicate that DOCK2 is a major Rac-GEF and is critical for the migration and activation of leukocytes, including neutrophils (16–18). In addition, bi-allelic loss-of-function mutations in *DOCK2* cause severe combined immunodeficiency in humans (19, 20). Thus, DOCK2 plays essential roles in immune surveillance mechanisms.

Cholesterol sulfate (CS) is a sulfated derivative of cholesterol and is widely distributed in various tissues and body fluids (21). In humans and mice, cholesterol sulfation is mediated primarily by the sulfotransferases SULT2B1b and, to a lesser extent, SULT2B1a, which are produced from the same gene, *SULT2B1*, through alternative splicing (22, 23). Currently, CS has been implicated in many biological processes including sperm capacitation, platelet adhesion, blood clotting, cholesterol or leukotriene biosynthesis, and T-cell receptor signaling (21, 24, 25). In addition, we have revealed that CS is an endogenous inhibitor of DOCK2 (26). It directly binds to the catalytic DHR-2 domain of DOCK2 and inhibits its Rac-GEF activity, suppressing immune cell migration (26). This inhibitory effect is CS-specific and has not been observed with other cholesterol derivatives (26).

This study aimed to elucidate the effect of SULT2B1-mediated CS production on immune responses in the intestinal tract. Through mass spectrometry (MS) analyses, we examined CS levels and localization in the SI and colon. Further, we investigated the functional role of CS during mucosal injury using *Sult2b1*-deficient (*Sult2b1^−/−^*) mice under dextran sodium sulfate (DSS)-induced colitis and indomethacin (IND)-induced SI ulcer formation. Our findings revealed a novel mechanism that prevents intestinal infiltration by immune cells.

## Materials and methods

### Mice

*Sult2b1-P2A-EGFP* knock-in mice were developed by using the CRISPR/Cas9 genome editing system. A targeting site within the exon 7 of mouse *Sult2b1* was selected using the CRISPRdirect web server (http://crispr.dbcls.jp/) (27). Single guide RNA (sgRNA) was transcribed *in vitro*, and a donor vector was constructed. Cas9, sgRNA, and the donor vector were microinjected into fertilized eggs of C57BL/6J mice. Fertilized eggs were implanted to obtain F0-positive mice and successful implantation was confirmed using PCR and sequencing. F0-positive mice were mated with C57BL/6J mice to generate stable F1-generation mice. *Sult2b1^−/−^* mice were obtained from the Jackson Laboratory (stock no. 018773; Bar Harbor, ME, USA). *Dock2^−/−^* mice have been previously described (11). *Sult2b1^−/−^* and *Dock2^−/−^* mice had been backcrossed with C57BL/6J mice more than 10 generations before use. C57BL/6J mice were purchased from CLEA Japan (Tokyo, Japan). The age-matched male mice (*Sult2b1^+/+^*, *Sult2b1^+/−^*, and *Sult2b1^−/−^* littermates) were used at 9–12 weeks of age. All mice were maintained under specific-pathogen-free conditions at the animal facility of Kyushu University. All animal experiments were conducted according to the relevant national and international guidelines described in the Act on Welfare and Management of Animals (Ministry of Environment of Japan) and Regulation of Laboratory Animals (Kyushu University) guidelines. The Ethics Committee on Animal Experiments at Kyushu University approved all the animal experiments performed in this study.

### DSS-induced colitis model

Mice were treated with 1.5−2.5% DSS (molecular weight, 36−50 kDa, MP Biomedicals, Solon, OH, USA) dissolved in drinking water for 5 or 6 days, respectively, followed by treatment with normal drinking water until the end of the experiment. The animals were monitored for weight loss (0, none; 1, 1−5%; 2, 5−10%; 3, 10−20%; 4, > 20%), stool consistency (0, normal stool; 2, loose stool; 4, diarrhea), and hemoccult (0, normal; 2, hemoccult positive; 4, gross blood) during the experiments. The Luminol Reaction Experiment Kit (Wako, Osaka, Japan) was used to detect fecal occult blood, as described previously (28).

### Antibiotic treatment for intestinal bacteria depletion

Intestinal bacteria in the mice were depleted by administering a combination of antibiotics [500 μL per mouse; ampicillin (6.7 mg/mL; Sigma-Aldrich, St Louis, MO, USA), neomycin (6.7 mg/mL; Sigma-Aldrich), vancomycin (3.35 mg/mL; Wako), and metronidazole (6.7 mg/mL; Sigma-Aldrich)] by oral gavage three times a week. The combined antibiotics or sterile distilled water (vehicle control) were administered to the mice for 2 weeks prior to DSS or IND treatment. In the DSS-induced colitis model, antibiotics or water were orally administered until the mice were euthanized.

### Quantification and imaging of CS using MS

Mice were decapitated following isoflurane anesthesia; their intestinal tissues were freshly isolated, quick-frozen with liquid nitrogen, and stored at −80°C until analyses. The frozen samples were mixed with an internal standard (IS; deuterium-labeled CS; d7-CS) and homogenized in ice-cold methanol (500 μL) using a homogenizer (Finger Masher AM79330; Sarstedt, Nümbrecht, Germany). The supernatant was filtered using ultrafiltration devices (UltrafreeMCPLHCC; Human Metabolome Technologies, Yamagata, Japan), and the filtrate was directly analyzed using liquid chromatography-tandem MS (LC-MS/MS) for CS content. The triple-quadrupole MS equipped with an electrospray ionization (ESI) ion source (LCMS-8040; Shimadzu Corporation, Kyoto, Japan) was used in the negative-ESI and multiple reaction monitoring modes. The samples were resolved on the Mastro-C18 column (2.1 mm × 100 mm, 3-μm, Shimadzu GLC, Tokyo, Japan) by isocratic flow of mobile phase A (200 mM ammonium acetate) and mobile phase B (methanol) at a ratio of 1:9, flow rate of 0.4 mL/min, and column temperature of 40°C. CS and IS (d7-CS) signals were monitored by ion transitions at *m/z* 465.3 > 97 and 472.3 > 97, respectively. The absolute content of CS was calculated using peak area ratios of CS against IS.

The matrix-assisted laser desorption/ionization (MALDI)-linear ion trap MS (MALDI LTQ XL; Thermo Fisher Scientific, Waltham, MA, USA) and Ultraflextreme MALDI-TOF/TOF (Bruker Daltonics, Bremen, Germany) were used for MALDI imaging analysis of CS and d7-CS, as described previously (29). d7-CS was purchased from Sigma-Aldrich (#903752). Data were acquired on the TOF/TOF and LTQ instruments in negative reflectron mode or negative selected ion monitoring mode, respectively, with raster scans at a pitch distance of 30 μm. Image reconstructions of data obtained with the TOF/TOF instrument were performed using the FlexImaging 4.1 software (Bruker Daltonics), and data obtained with the LTQ instrument were performed using ImageQuest v.1.0.1 software (Thermo Fisher Scientific).

### Western blotting

Total cell lysates were prepared and separated using sodium dodecyl-sulfate polyacrylamide gel electrophoresis (SDS-PAGE), as previously described (30). Briefly, intestinal tissues were homogenized in a 1.5-mL tube containing 250 μL of 1× cell lysis buffer (#9803; CST, Danvers, MS, USA) supplemented with a cocktail of complete protease inhibitors (Roche, Basel, Switzerland) using an electric homogenizer for 1 min on ice. After centrifugation, supernatants were mixed with an equal volume of 2× sample buffer [125 mM Tris-HCl, 0.01% bromophenol blue, 4% SDS, 20% glycerol, and 200 mM dithiothreitol (DTT)] and boiled for 10 min. Total protein concentration was measured using the DC™ Protein Assay Reagent (Bio-Rad, Hercules, CA, USA). Tissue extracts were separated using SDS-PAGE and immunoblotted with the following antibodies: rabbit anti-SULT2B1b [custom-made (26); 1:1,000], and goat anti-β-actin (#sc-1616; 1:2,000, Santa Cruz Biotechnology, TX, USA). The following horseradish peroxidase-conjugated secondary antibodies were used: mouse anti-rabbit IgG (#sc-2357; 1:2,000, Santa Cruz Biotechnology), and mouse anti-goat IgG (#sc-2354; 1:2,000, Santa Cruz Biotechnology).

### Histology and immunohistochemistry

Colon tissues were fixed in 4% (w/v) paraformaldehyde (Wako) for 18 h at 4°C and embedded in paraffin blocks. Sections were stained using hematoxylin and eosin (H&E) and histological scores were assigned by a trained and blinded pathologist, as previously described (31). Eight pathological changes, including the extent of inflammatory cell infiltration, goblet cell reduction, decreased crypt density, crypt hyperplasia, thickening of the muscle layer, extent of submucosal tissue inflammation, crypt abscess, and ulceration, were rated on a 0–3 scale from normal to severe. The sum of each score (maximum 24) was used as the histological score. For immunofluorescence analyses, fixed tissues were incubated with 30% sucrose (Wako) in phosphate-buffered saline (PBS) for 18 h at 4°C and embedded in O.C.T. compound (Sakura Finetek, Osaka, Japan). After freezing at −80 °C, cryostat sections were blocked with G-Block (GenoStaff, Tokyo, Japan) for 15 min at room temperature. Samples were stained using Alexa Fluor™ 488-conjugated WGA overnight at 4°C. Nucleus staining was performed using 4′,6-diamidino-2-phenylindole (DAPI; Dojindo, Kumamoto, Japan). All images were obtained using a laser scanning confocal microscope (FV3000; Olympus).

### RNA isolation and real-time PCR analysis

Colon tissues were homogenized in TRIzol™ reagent (Thermo Fisher Scientific) on ice for 1 min using an electric homogenizer. Next, total RNA was extracted using the TRIzol™ Plus RNA Purification Kit (Thermo Fisher Scientific). The purity and concentration of RNA were assessed using the NanoDrop™ device (ND-1000; Thermo Fisher Scientific). RNA samples were reverse-transcribed using PCR, as previously described (30). Bacterial DNA was isolated from fecal samples using the QIAamp Fast DNA Stool Mini Kit (QIAGEN, Valencia, CA, USA). Real-time PCR was performed on a CFX Connect™ Real-Time PCR Detection System (Bio-Rad) using SYBR Green PCR Master Mix (Thermo Fisher Scientific). Target gene expression was normalized to that of *Hprt*. Primer sequences are listed in **Supplementary Table 1**. *Bacteroides* spp. and total bacteria in fecal samples were detected using the following primer pairs for 16S rRNA (32): *Bacteroides* (5′-GAGAGGAAGGTCCCCCAC-3′ and 5′-CGCTACTTGGCTGGTTCAG-3′) and total bacteria (*Eubacteria*: 5′-CGGTGAATACGTTCCCGG-3′ and 5′-TACGGCTACCTTGTTACGACTT-3′). Melt curve analysis was performed to ensure specificity of the amplification products.

### IL-6 and S100A8 enzyme-linked immunosorbent assay

Mice were euthanized by decapitation and blood samples were obtained after a cardiac puncture to measure the plasma IL-6 concentration using the Mouse IL-6 ELISA kit (Thermo Fisher Scientific). To quantify S100A8 in feces, fecal samples (2–3 feces per mouse) were resuspended in cold fecal protein extraction buffer (50 mM Tris, pH 7.5, 150 mM NaCl). The samples were homogenized and incubated on ice for 30 min, briefly vortexed every 5 min, and then centrifuged at 2000 × *g* at 4 °C for 10 min. The supernatants were diluted to 1:10 using PBS. The fecal concentration of S100A8 was measured using the Mouse S100A8 DuoSet ELISA kit (R&D Systems, Minneapolis, MN, USA).

### CyTOF and flow cytometry

Intraepithelial and lamina propria cells were isolated using the Lamina Propria Dissociation Kit (Myltenyi Biotec, Bergishe Gladbach, Germany), according to the manufacturer’s instructions. Briefly, the colon or SI was washed with PBS and diced. Tissues were incubated with 1× Hanks’ Balanced Salt Solution (HBSS) containing 5 mM ethylenediaminetetraacetic acid (Sigma-Aldrich), 5% fetal calf serum (Thermo Fisher Scientific), and 1 mM DTT (Sigma-Aldrich) for 20 min at 37 °C with continuous rotation using the MACSmix Tube Rotator (Myltenyi Biotec). After vortexing for 10 s, tissues were passed through a 100-μm MACS SmartStrainer (Myltenyi Biotec). The flow-through containing intraepithelial cells was collected and viable cells were recovered using density gradient centrifugation with the Lympholyte-M Cell Separation Media (Cedarlane, Hornby, Ontario, Canada). The lamina propria tissue samples were transferred into a fresh tube and incubated for 30 min at 37 °C with the Lamina Propria Dissociation Kit and gentleMACS™ Dissociators (Myltenyi Biotec). Cell suspensions were stained for viability using Cell-ID Intercalator-103Rh (1:500, Standard BioTools, South San Francisco, CA, USA) and blocked with anti-mouse CD16/32 antibody (1:1000, 2.4G2, TONBO Biosciences, San Diego, CA, USA) for 10 min prior to staining with metal-conjugated antibodies (**Supplementary Table 2**); cells were then prepared, as previously described (33), and acquired on a Helios CyTOF Mass Cytometer (Standard BioTools). CD45^+^ live singlets were subjected to viSNE analysis using Cytobank Premium (Cytobank Inc., Santa Clara, CA, USA).

For flow cytometry, intraepithelial and lamina propria cells were prepared as described above. Cells were incubated for 10 min at room temperature with Fixable Viability Stain 510 (BD Biosciences, San Jose, CA, USA), washed, and re-incubated for 10 min on ice with anti-mouse CD16/32 (TONBO Biosciences) to block the Fc receptors. The cells were subsequently stained with the following antibodies: anti-mouse CD45 (1:100, 30-F11, BioLegend, San Diego, CA, USA), anti-mouse CD11b (1:100, M1/70, BD Biosciences), and anti-mouse Gr-1 (1:100, RB6-8C5, TONBO Biosciences). Flow cytometric analysis was performed with the BD FACSVerse™ equipped with BD FACSuite™ software (BD Biosciences).

### ROS production of neutrophils

Mouse BM-derived neutrophils were isolated from femurs and tibias of mice using HBSS (Thermo Fisher Scientific) containing 0.5% bovine serum albumin (BSA; Sigma-Aldrich). Cells were resuspended with 0.5 mL HBSS containing 0.5% BSA and treated under hypotonic conditions for 10 s by adding 3 mL of sterile distilled water, and then supplemented with 0.3 mL of 10% NaCl to restore the osmolarity. Cells were layered on a discontinuous Percoll (Sigma-Aldrich) gradient. After centrifugation, cells at the 62/81% interface were recovered and washed with HBSS and then with RPMI (Wako). Cells were resuspended in RPMI containing 2 μL of luminol (50 mg/mL; Wako) with 12.5 μM CS (#C9523; Sigma-Aldrich), 1.54 μM SOD (Sigma-Aldrich), or vehicle (0.2% dimethyl sulfoxide; Wako) at 37°C for 60 min. Cells were stimulated with 100 nM PMA (Sigma-Aldrich) and luminol luminescence due to nicotinamide adenine dinucleotide phosphate activity was measured using an IVIS Imaging System (IVIS Spectrum; PerkinElmer, Waltham, MA, USA) at each time point.

### Circulating neutrophil depletion with anti-Ly6G antibody

Neutrophil depletion in blood was performed, as described previously (34). Anti-Ly6G (1A8, #BP0075-1, BioXCell, West Lebanon, NH, USA), anti-rat Kappa immunoglobulin (MAR 18.5, #BE0122, BioXCell), and the corresponding isotype control (2A3, #BP0089, BioXCell) were injected intraperitoneally into the mice.

### Oral gavage of CS and IND-induced SI injury

To induce SI injury, IND (Wako) dissolved in 5% NaHCO_3_ was subcutaneously administered in the back neck of the mice (5 mg/kg body weight), as described previously (35). CS (200 mg/kg body weight; Sigma-Aldrich) or vehicle alone was orally administered to mice three times at 4 h intervals. d7-CS (330 μg per mouse; Sigma-Aldrich) was orally administered to *Sult2b1^+/+^* mice and assessed 4 h later. CS and d7-CS were dissolved in 40 mM 2-hydroxypropyl-beta-cyclodextrin (HPβCD; Sigma-Aldrich) before use. Twenty-four hours after IND administration, mice were intravenously administered 150 μL of 0.4% Evans blue dye (Wako) dissolved in PBS, and the mice were euthanized by decapitation 30 min later. The number of ulcer lesions in the SI stained with blue dye were counted under the stereomicroscope (SZ-PT; Olympus) and these areas were quantified using Image J (National Institutes of Health, Bethesda, MD, USA). Ulcer lesions in the SI were also sampled for flow cytometry analysis to calculate the absolute number of neutrophils per wet weight.

### Statistical analyses

Graphing and statistical analyses were performed using Prism 8 (GraphPad Software, La Jolla, CA). The data were initially tested using a Kolmogorov–Smirnov test for normal distribution. For comparison between the two groups, parametric and nonparametric data were analyzed using a two-tailed unpaired Student’s *t*-test and a two-tailed Mann–Whitney test, respectively. Statistical differences between more than two experimental groups were evaluated using analysis of variance (ANOVA) with Dunnett’s multiple comparison test. Survival analysis was performed using Kaplan–Meier curves, with comparisons between groups made using the log-rank test. Data are expressed as mean ± standard deviation (SD), and *P*-values of less than 0.05 were considered significant. All sample sizes and statistical tests employed are documented in figure legends.

## Results

### Endogenous CS is produced at the mucosal-luminal interface

**Figure 1:**
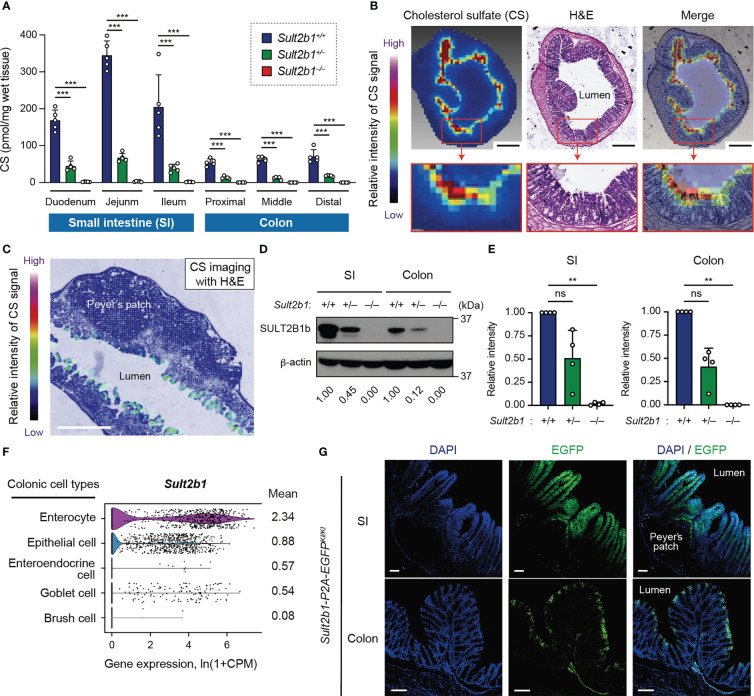
Endogenous CS is produced at the mucosal-luminal interface. **(A)** The amount of CS in the SI and colon of *Sult2b1^+/+^*, *Sult2b1^+/−^*, and *Sult2b1^−/−^* mice were quantified using liquid chromatography-tandem mass spectrometry (LC-MS/MS; n = 5 mice per group; one-way ANOVA with Dunnett’s multiple comparison test). **(B, C)** Localization of CS in sections of the colon and SI in *Sult2b1^+/+^* mice visualized using hematoxylin and eosin (H&E) staining and MS imaging. Color bar indicates the relative intensities of the CS signal [mass/charge ratio (*m/z*) 465]. Scale bar, 500 μm. **(D, E)** Representative immunoblots showing SULT2B1b in the SI and colon. The numbers below the blot indicate the abundance of SULT2B1b relative to β-actin for each tissue and are normalized considering the abundance of SULT2B1b in *Sult2b1^+/+^* mice as 1. Bar graphs showing the quantification data of four individual blots for each tissue (n = 4; one-way ANOVA with Dunnett’s multiple comparison test). **(F)** Violin plots showing mouse *Sult2b1* gene expression in each colonic cell type, analyzed using the *Tabula Muris* single-cell RNA-seq data (3-month-old C57BL/6JN mice). Data for gene counts from cells sorted using flow cytometry were normalized to counts per million (CPM) and presented as l_n_(1 + CPM). The numbers next to the plots indicate mean expression of *Sult2b1* in each cell type. **(G)** Representative images showing the SI and colon of *Sult2b1-P2A-EGFP* mice counterstained with 4′,6-diamidino-2-phenylindole (DAPI). Scale bar, 100 μm. Data were obtained from two **(A-C)**, four **(D, E)**, and three **(G)** independent experiments, and graphs are shown as the mean ± standard deviation (SD). **P < 0.01; ***P < 0.001; ns, not significant.

We have previously found that the SI expresses SULT2B1 and produces CS in mice (26). To quantify CS produced in the intestinal tract, we measured CS levels in whole tissues of the SI and colon among *Sult2b1^+/+^*, *Sult2b1^+/−^*, and *Sult2b1^−/−^* mice using MS analysis. Although CS was absent throughout the intestinal tract of *Sult2b1^−/−^* mice, it was produced in the duodenum, jejunum, ileum, and the whole colon of *Sult2b1^+/+^* mice (**Figure 1A**). Moreover, MS analysis revealed markedly decreased CS levels, even in *Sult2b1^+/−^* mice (**Figure 1A**). Recent microbiological studies have reported that *Bacteroides* spp. produce CS in humans and mice *via* a specific enzyme with sulfotransferase-like activity (36, 37). Consistently, CS and *Bacteroides* spp. were detected in the feces of *Sult2b1^−/−^* mice (**Supplementary Figures 1A, B**), indicating that intestinal CS was produced by both intestinal tissues and commensal bacteria. However, CS concentration in feces collected from *Sult2b1^−/−^* mice was markedly reduced to 21% of that from *Sult2b1^+/+^* mice, even though *Bacteroides* spp. increased in the gut of *Sult2b1^−/−^* mice (**Supplementary Figures 1A, B**). These findings suggest that IECs are the primary source of intestinal CS.

To identify CS-producing regions in the intestinal tract, we further investigated CS localization using MS imaging. CS was specifically concentrated at the top of the villi close to the intestinal lumen in the SI and colon of *Sult2b1^+/+^* mice (**Figures 1B, C**). In contrast, CS production was not detected in *Sult2b1^−/−^* mice (**Supplementary Figure 1C**). Cholesterol sulfation is mediated by SULT2B1b produced from the gene *Sult2b1* in mice and *SULT2B1* in humans (22, 23, 26). Western blot analyses showed the presence of SULT2B1b in the SI and colon of *Sult2b1^+/+^* mice (**Figure 1D**). On the other hand, SULT2B1b expression levels in *Sult2b1^+/−^* mice were reduced to 51 and 41% of that in *Sult2b1^+/+^* mice in the SI and colon, respectively (**Figures 1D, E**), demonstrating that gene dosage affects SULT2B1b expression. The publicly available single-cell RNA-seq data [*Tabula Muris* (38)] revealed upregulation of the *Sult2b1* gene in the enterocytes of the colon tissue, which are nutrient-absorbing intestinal epithelial cells (IECs; **Figure 1F** and **Supplementary Figure 1D**). Additionally, we generated *Sult2b1-P2A-EGFP* knock-in mice (**Supplementary Figure 2**) and confirmed the presence of *Sult2b1*-expressing enhanced green fluorescent protein (EGFP)^+^ cells in the upper villi of the SI and colon (**Figure 1G**). Therefore, IEC-derived CS may have physiological roles at the interface between the intestinal lumen and mucosa.

### CS-deficient mice exhibit severe colitis during DSS-induced mucosal injury

**Figure 2:**
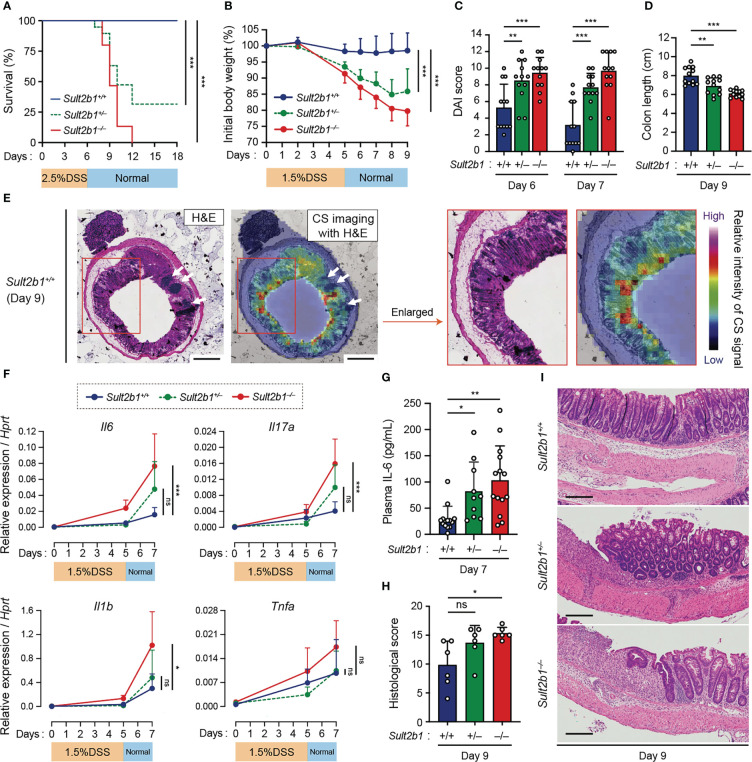
CS-deficient mice exhibit severe colitis during DSS-induced mucosal injury. **(A)** Kaplan–Meier cumulative survival curve of *Sult2b1^+/+^*, *Sult2b1^+/−^*, and *Sult2b1^−/−^* mice treated with 2.5% DSS in drinking water for 6 days (n = 15, 19, and 15 mice per group, respectively; log-rank test). **(B–D)** Body weights, DAI scores, and colon lengths of *Sult2b1^+/+^*, *Sult2b1^+/−^*, and *Sult2b1^−/−^* mice treated with 1.5% DSS in drinking water for 5 days (n = 12 mice per group; one-way ANOVA with Dunnett’s multiple comparison test). **(E)** CS localization in the colon cross-section from *Sult2b1^+/+^* mice visualized using H&E staining and MS imaging. Color bar indicates the relative intensities of the CS signal (*m/z* 465). White arrows indicate solitary intestinal lymphoid tissues. Enlarged (boxed) areas are shown on the right. Scale bar, 500 μm. **(F)** Real-time PCR analysis of inflammatory gene expression in the entire colon on day 0, 5, and 7 after a 5-day 1.5% DSS challenge (n = 7 mice per group; one-way ANOVA with Dunnett’s multiple comparison test). Target gene expression was normalized to *Hprt* expression. **(G–I)** Plasma concentrations of IL-6, histological scores, and the representative H&E staining of colon sections from 1.5% DSS-treated mice on days 7 and 9 (n = 7 mice per group; one-way ANOVA with Dunnett’s multiple comparison test). Scale bar, 200 μm. Data were obtained from four **(A)**, three (**B–D** and **F–I**), and two **(E)** independent experiments, and graphs are shown as the mean ± SD. *P < 0.05; **P < 0.01; ***P < 0.001; ns, not significant.

To examine whether CS affects gut inflammation, we compared the severity of DSS-induced colitis among *Sult2b1^+/+^*, *Sult2b1^+/−^*, and *Sult2b1^−/−^* mice. DSS-induced colitis is widely practiced, owing to several similarities with human UC (39). Macroscopic analyses of the colon, SI, and spleen revealed no significant difference between *Sult2b1^+/+^* and *Sult2b1^−/−^* mice at steady state (**Supplementary Figure 3**). However, on treating these mice with 2.5% DSS for 6 days, the survival rate on day 18 was markedly reduced in the absence of *Sult2b1* (**Figure 2A**: *Sult2b1^+/+^* mice, 100%; and *Sult2b1^−/−^* mice, 0%). Consistent with the CS production levels (**Figure 1A**), the survival rate of *Sult2b1^+/−^* mice was also reduced to 32% (**Figure 2A**).

Based on these findings, we speculated that even short exposures to and/or low concentrations of DSS would induce severe colitis in *Sult2b1^+/−^* and *Sult2b1^−/−^* mice. To test this hypothesis, we treated *Sult2b1^+/+^*, *Sult2b1^+/−^*, and *Sult2b1^−/−^* mice with 1.5% DSS for 5 days. *Sult2b1^+/−^* and *Sult2b1^−/−^* mice exhibited exacerbated weight loss and increased disease activity index (DAI) scores, compared to that in *Sult2b1^+/+^* mice (**Figures 2B, C**). Consistently, on day 9, the colon length in *Sult2b1^+/−^* and *Sult2b1^−/−^* mice was shorter than that in *Sult2b1^+/+^* mice (**Figure 2D**). After the DSS challenge, CS diffused throughout the crypts from the luminal side toward the muscularis mucosae (**Figure 2E**). Although the gene expression levels of inflammatory cytokines including interleukin (*Il)6*, *Il17a*, *Il1b*, and tumor necrosis factor-α (*Tnfa*) gradually increased with colitis progression in *Sult2b1^+/+^* mice, these levels were considerably higher in *Sult2b1^+/−^* and *Sult2b1^−/−^* mice than those in *Sult2b1^+/+^* mice on day 7 (**Figure 2F**). Similarly, plasma IL-6 levels on day 7 increased in *Sult2b1^+/−^* and *Sult2b1^−/−^* mice, compared to those in *Sult2b1^+/+^* mice (**Figure 2G**). Moreover, histological analysis revealed severe colitis in *Sult2b1^−/−^* mice, characterized by immune cell infiltration, goblet cell loss, and ulceration (**Figures 2H, I**). Thus, the lack of CS exacerbated DSS-induced colitis.

### Lack of CS does not exacerbate colitis in the absence of intestinal bacteria or by the genetic deletion of *Dock2*

**Figure 3:**
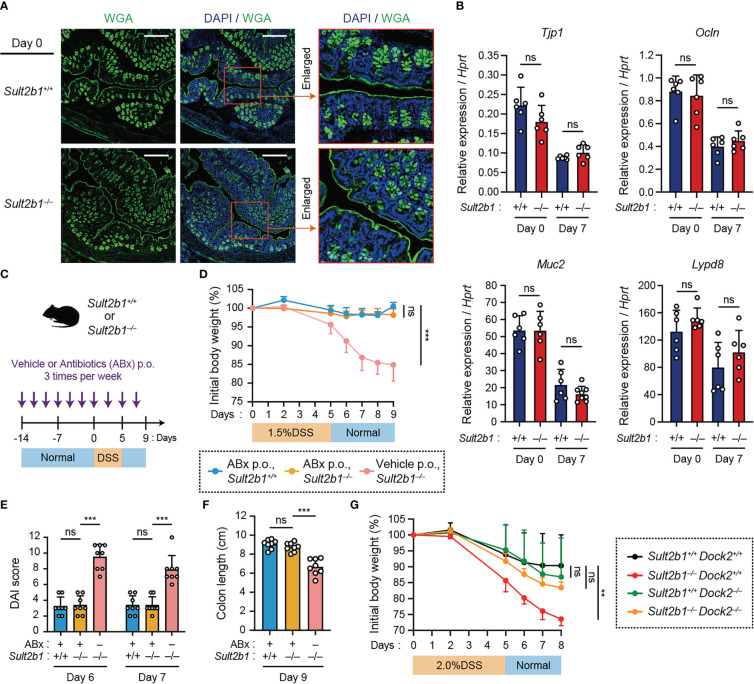
Lack of CS does not exacerbate colitis in the absence of intestinal bacteria or by the genetic deletion of *Dock2.* **(A)** Representative images showing the colon tissue of *Sult2b1^+/+^* mice (top) and *Sult2b1^−/−^* mice (bottom) at steady state. Samples were stained using Alexa Fluor™ 488-conjugated wheat germ agglutinin (green) and DAPI (nucleus; blue). Scale bar, 200 μm. **(B)** Real-time PCR analysis of the indicated gene expression in the entire colon of *Sult2b1^+/+^* and *Sult2b1^−/−^* mice on days 0 and 7 after a 5-day 1.5% DSS challenge (n = 7 mice per group; two-tailed unpaired Student’s *t*-test). Target gene expression was normalized to *Hprt* expression. **(C)** Schematic illustration of the protocol used for antibiotic treatment. A mixture of several antibiotics (ABx) or vehicle was orally administered to mice before and during the DSS challenge. **(D–F)** Body weights, DAI scores, and colon lengths of *Sult2b1^+/+^* and *Sult2b1^−/−^* mice administrated ABx or vehicle and 1.5% DSS in drinking water for 5 days (n = 8 mice per group; one-way ANOVA with Dunnett’s multiple comparison test). **(G)** Body weights of *Sult2b1^+/+^Dock2^+/+^*, *Sult2b1^−/−^Dock2^+/+^*, *Sult2b1^+/+^Dock2^−/−^*, and *Sult2b1^−/−^Dock2^−/−^* mice treated with 2.0% DSS in drinking water for 5 days (n = 6 mice per group; one-way ANOVA with Dunnett’s multiple comparison test). Data were obtained from three independent experiments **(A-G)**, and graphs are shown as the mean ± SD. **P < 0.01; ***P < 0.001; ns, not significant.

To elucidate the functional role of CS, we examined whether it affects the barrier function of IECs *in vivo*. Staining intestinal sections with wheat germ agglutinin (WGA), a lectin bound to mucins on the luminal surface epithelium and in goblet cells, revealed similar mucin distribution between *Sult2b1^+/+^* and *Sult2b1^−/−^* mice (**Figure 3A**). In addition, mRNA expression levels of *Tjp1*, *Ocln*, *Muc2*, and *Lypd8*, which are critical for IEC barrier functions (40–42), were comparable between *Sult2b1^+/+^* and *Sult2b1^−/−^* mice at steady state and after 1.5% DSS challenge (**Figure 3B**). Therefore, CS function may not be mediated by enhancing intestinal barrier function. We further determined whether CS-deficient mice exhibit severe colitis in the absence of commensal bacteria during DSS-induced mucosal injury. By bacterial depletion using oral antibiotics prior to the 1.5% DSS challenge (**Figure 3C**), the clinical signs of colitis, such as body weight loss, DAI score, and colon length shortening, were recovered in *Sult2b1^−/−^* mice to the levels similar to those in *Sult2b1^+/+^* mice (**Figures 3D–F**). Thus, these results showed that the lack of CS does not exacerbate colitis in the absence of intestinal bacteria.

Then, we hypothesized that DOCK2 in immune cells is the functional target of CS during colitis because CS binds to the catalytic domain of DOCK2 and inhibits its Rac-GEF activity (26). To address this hypothesis, we crossed *Sult2b1^−/−^* with *Dock2^−/−^* mice to obtain *Sult2b1* and *Dock2* double knockout mice (*Sult2b1^−/−^Dock2^−/−^*). When mice were treated with 2.0% DSS for 5 days, *Sult2b1^−/−^Dock2^+/+^* mice exhibited exacerbated weight loss compared to *Sult2b1^+/+^Dock2^+/+^* mice (**Figure 3G**). In contrast, there was no difference among *Sult2b1^+/+^Dock2^+/+^*, *Sult2b1^+/+^Dock2^−/−^*, and *Sult2b1^−/−^Dock2^−/−^* mice in the degree of weight loss (**Figure 3G**), indicating that CS alleviates DSS-induced colitis by inhibiting DOCK2.

### CS deficiency alters the composition of immune cells during gut inflammation

**Figure 4:**
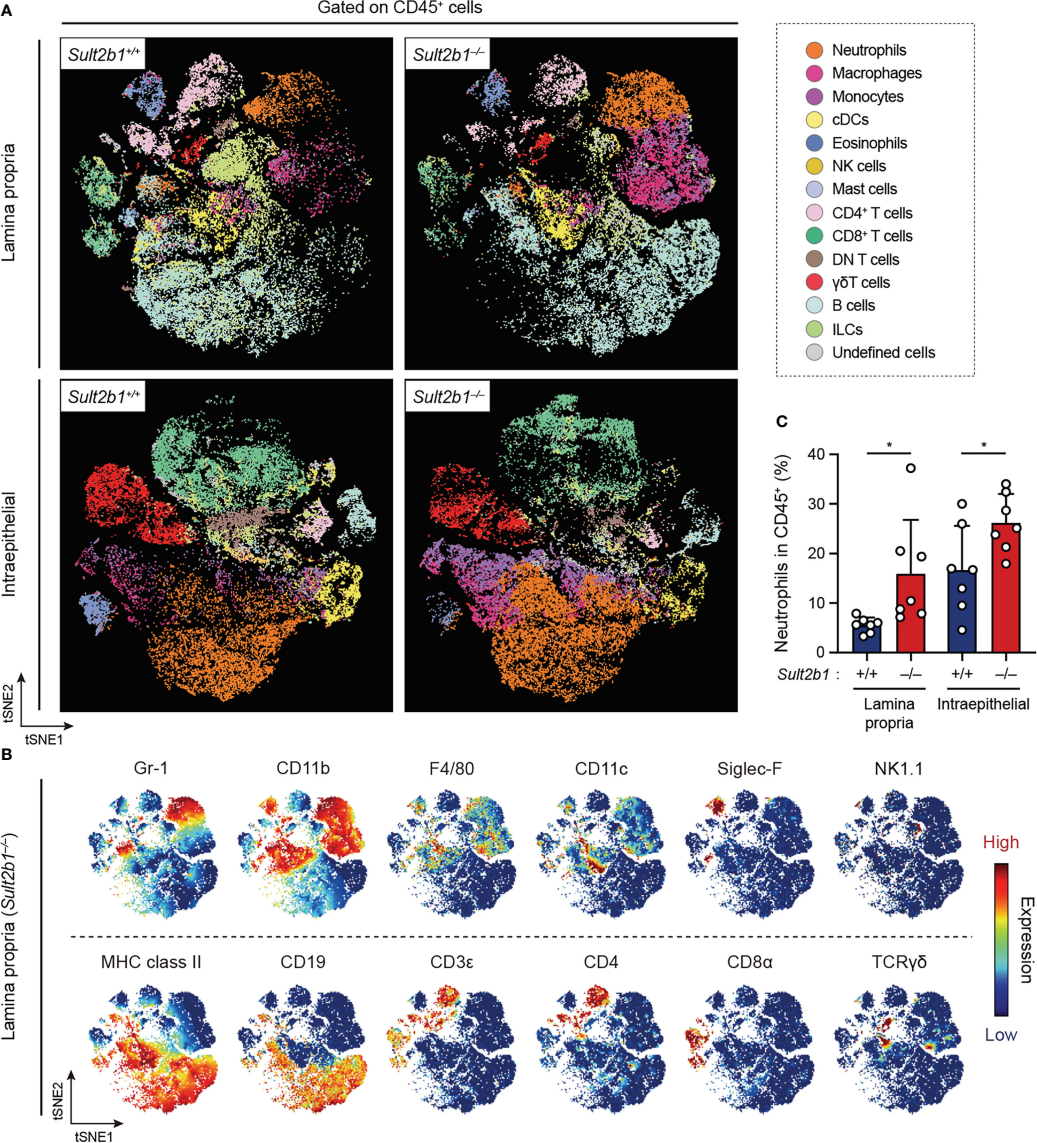
CS deficiency alters the composition of immune cells during gut inflammation. **(A)** FlowSOM analyses of intraepithelial and lamina propria immune cells isolated from the colon of *Sult2b1^+/−^* and *Sult2b1^−/−^* mice on day 7 after a 5-day 1.5% DSS challenge. After gating on singlets, live CD45^+^ cells were concatenated from 7 mice per group and clustered using the viSNE. Distinct metaclusters are shown in different colors. **(B)** t-SNE plots overlaid with the expression heatmaps of individual markers (red and blue indicate high and low expression, respectively). **(C)** Percentage of neutrophils (CD45^+^ CD3ε^−^ CD19^−^ Gr-1^+^ CD11b^+^) to the total intraepithelial and lamina propria CD45^+^ cells (n = 7 mice per group; two-tailed unpaired Student’s *t*-test). Data were obtained from three independent experiments **(A-C)**, and graphs are shown as the mean ± SD. *P < 0.05.

To comprehensively analyze the immune cell profiles in the colon from *Sult2b1^+/+^* and *Sult2b1^−/−^* mice during DSS-induced colitis, we performed high-dimensional phenotyping of intraepithelial and lamina propria immune cells using cytometry by time-of-flight (CyTOF). After gating on singlets and live CD45^+^ cells, colonic immune cells were subdivided into 14 populations (**Figure 4A**), which were identified by the differential expression of individual lineage markers and the visualization of t-distributed stochastic neighbor embedding (viSNE) algorithms (**Figure 4B**). On day 7 after the 1.5% DSS challenge, CyTOF analyses revealed increased proportions of neutrophils, monocytes, and macrophages in the lamina propria and intraepithelial CD45^+^ cells of *Sult2b1^−/−^* mice, compared to those in *Sult2b1^+/+^* mice (**Figure 4C** and **Supplementary Figure 4**). In contrast, the proportions of conventional dendritic cells, eosinophils, natural killer cells, and CD8α^+^ T cells decreased in *Sult2b1^−/−^* mice, with no significant difference in the percentage of mast cells, CD4^+^ T cells, γδ T cells, and B cells (**Supplementary Figure 4**). Thus, CS deficiency altered the composition of immune cells during DSS-induced mucosal injury.

### Depletion of circulating neutrophils leads to the alleviation of colitis in *Sult2b1^−/−^* mice

**Figure 5:**
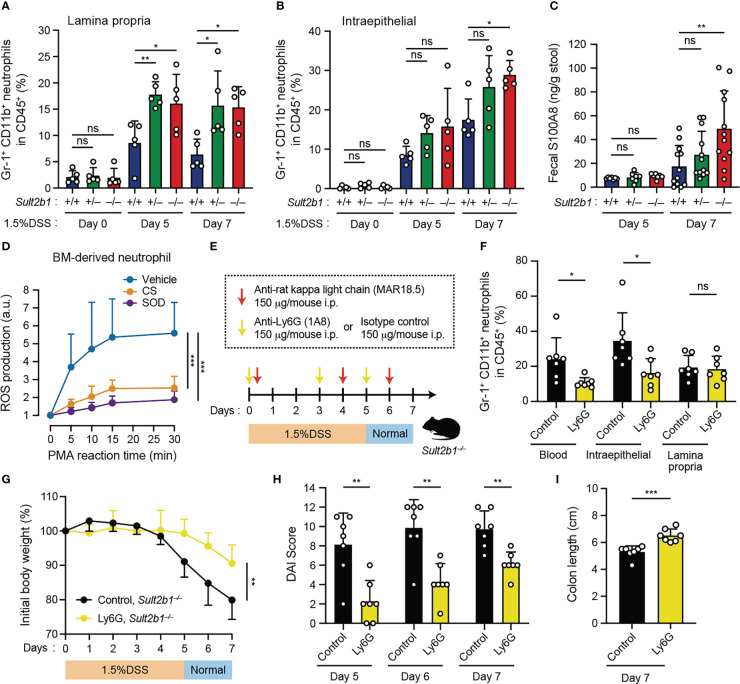
Depletion of circulating neutrophils leads to the alleviation of colitis in *Sult2b1^−/−^* mice. **(A, B)** The percentage of CD11b^+^ Gr-1^+^ neutrophils in lamina propria CD45^+^ cells or intraepithelial CD45^+^ cells in the colon from *Sult2b1^+/+^*, *Sult2b1^+/−^*, and *Sult2b1^−/−^* mice (n = 5 mice per group; one-way ANOVA with Dunnett’s multiple comparison test). **(C)** Fecal concentrations of S100A8 in 1.5% DSS-treated mice on days 5 and 7 (n = 12 mice per group; one-way ANOVA with Dunnett’s multiple comparison test). **(D)** ROS production in wild-type bone marrow-derived neutrophils stimulated using PMA (100 nM). Data are presented as the ratio after normalization of the 0-min value to an arbitrary unit (a.u.) of 1 (n = 6 mice per group; one-way ANOVA with Dunnett’s multiple comparison test). **(E)** Schematic illustration of the protocol used for circulating neutrophil depletion. Indicated antibodies were intraperitoneally administrated to *Sult2b1^−/−^* mice. **(F)** The percentage of CD11b^+^ Gr-1^+^ neutrophils in CD45^+^ cells of the blood and the colon tissues from *Sult2b1^−/−^* mice after antibody treatments (n = 7 mice per group; two-tailed unpaired Student’s *t*-test). **(G-I)** Body weights, DAI scores, and colon length of anti-Ly6G-treated or isotype control-treated *Sult2b1^−/−^* mice (n = 7 mice per group; two-tailed unpaired Student’s *t*-test). Data were obtained from five **(A, B)** and three **(C-I)** independent experiments, and graphs are shown as the mean ± SD. *P < 0.05; **P < 0.01; ***P < 0.001; ns, not significant.

It has been shown that neutrophils are commonly involved in several inflammatory diseases through infiltration into the tissue and ROS production (43). Therefore, we focused on neutrophils and compared their prevalence in the colon of *Sult2b1^+/+^*, *Sult2b1^+/−^*, and *Sult2b1^−/−^* mice by using flow cytometry. We found that, irrespectively of *Sult2b1* expression, the percentage of CD45^+^ cells in the total live cells gradually increased in the colon during the progression of colitis; however, the percentages of CD45^+^ cells were unchanged among these mice (**Supplementary Figures 5A, B**). In contrast, the neutrophil prevalence in CD45^+^ cells of the lamina propria was considerably higher in *Sult2b1^+/−^* and *Sult2b1^−/−^* mice than that in *Sult2b1^+/+^* mice on days 5 and 7 (**Figure 5A** and **Supplementary Figure 5C**). Similarly, on day 7, the prevalence of intraepithelial neutrophils in CD45^+^ cells increased in *Sult2b1^−/−^* mice compared to that in *Sult2b1^+/+^* mice (**Figure 5B** and **Supplementary Figure 5C**). During colitis, neutrophils abundantly express and secrete S100A8 protein, which forms an S100A8/S100A9 heterodimer (also called calprotectin; a non-invasive biomarker in IBD) (44). As expected, *Sult2b1^−/−^* mice exhibited a notably higher fecal level of S100A8 protein than that in *Sult2b1^+/+^* mice on day 7 after the DSS challenge (**Figure 5C**). Furthermore, we evaluated whether CS could inhibit reactive oxygen species (ROS) production in neutrophils, because ROS production by murine and human neutrophils largely depends on DOCK2-mediated Rac activation (18, 20). When wild-type bone marrow (BM)-derived neutrophils were stimulated with phorbol myristate acetate (PMA), they produced ROS in a superoxide dismutase (SOD)-inhibitable manner, and this ROS production was also inhibited by CS treatment (**Figure 5D**). These results suggest that CS could suppress infiltration of and ROS production by neutrophils in the intestinal tract.

As neutrophil recruitment was enhanced in CS-deficient mice after the DSS challenge, we assessed the contribution of circulating neutrophils to colitis using anti-Ly6G neutralizing antibodies. Based on the results of a previously reported neutrophil depletion protocol with some modifications (34), we confirmed that circulating neutrophils were reduced in the blood (**Figures 5E, F**). Although neutrophils in the lamina propria were not reduced (**Figure 5F**), anti-Ly6G-treated *Sult2b1^−/−^* mice exhibited significant recovery of the clinical signs including body weight loss, increased DAI scores, and colon length shortening, compared to isotype control-treated *Sult2b1^−/−^* mice (**Figures 5G-I**). Thus, these results indicate that reducing the number of circulating neutrophils leads to the alleviation of DSS-induced colitis in *Sult2b1^−/−^* mice.

### CS ameliorates IND-induced ulcers in the SI

Although NSAIDs are one of the most commonly used drugs owing to their analgesic properties, their adverse effects, such as gastrointestinal tract ulcers, are concerning (9, 10). NSAID-induced gastric ulcers can be controlled by consuming acid-inhibitory drugs; no medicines can prevent NSAID-induced SI ulcers (9, 10). As IECs in the SI expressed *Sult2b1* and produced CS abundantly (**Figure 1**), CS might also act in the SI to alleviate excessive inflammation.

**Figure 6:**
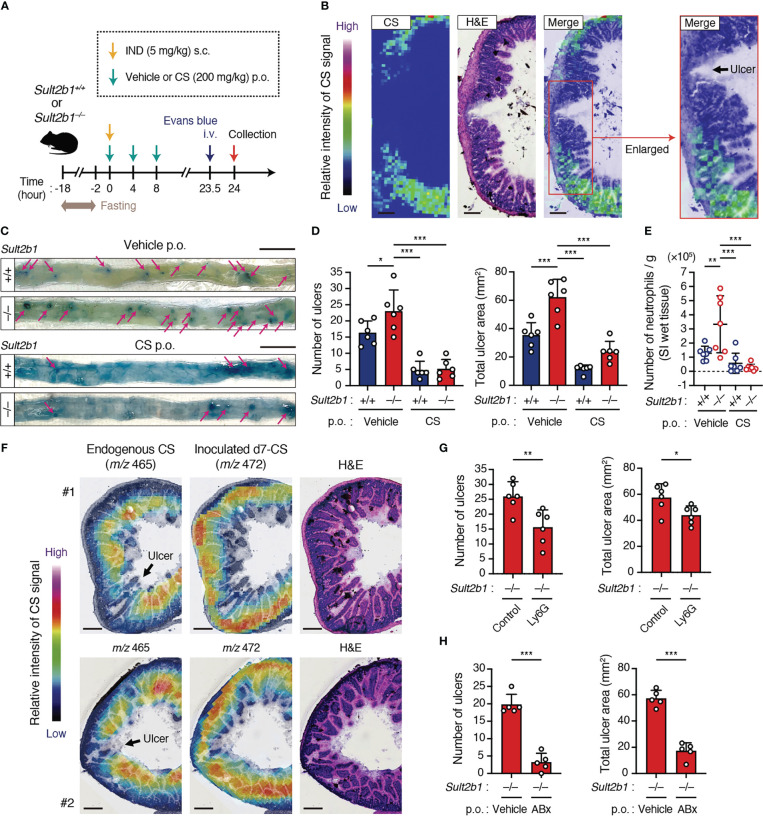
CS ameliorates IND-induced ulcers in the SI. **(A)** Schematic illustration of the protocol used for the IND-induced ulcer model and CS treatment. Vehicle or CS was orally administrated to mice three times at 4-hour intervals. **(B)** Localization of CS in the cross-section of the SI from IND-treated *Sult2b1^+/+^* mice visualized using MS imaging and H&E staining. The color bar indicates the relative intensities of the CS signal. The enlarged (boxed) area is shown on the right. Scale bar, 200 μm. **(C)** Representative macroscopic images of IND-induced SI ulcers in *Sult2b1^+/+^* and *Sult2b1^−/−^* mice after vehicle or CS administration. Ulcerative lesions (magenta arrows) are recognized as blue spots in the SI. Scale bar, 10 mm. **(D)** The number of ulcers (left) and total ulcer area (right) in the SI of *Sult2b1^+/+^* and *Sult2b1^−/−^* mice after IND injection and CS administration (n = 6 mice per group; one-way ANOVA with Dunnett’s multiple comparison test). **(E)** The absolute number of CD45^+^ CD11b^+^ Gr-1^+^ neutrophils per 100-mg SI (wet tissue) of *Sult2b1^+/+^* and *Sult2b1^−/−^* mice after IND injection and CS administration (n = 7 mice per group; one-way ANOVA with Dunnett’s multiple comparison test). **(F)** Localization of endogenous CS (*m/z* 465) and inoculated d7-CS (*m/z* 472) in the cross-section of the SI from IND-treated *Sult2b1^+/+^* mice visualized using MS imaging and H&E staining. The color bar indicates the relative intensities of the CS signal. Scale bar, 200 μm. **(G, H)** The number of ulcers (left) and total ulcer area (right) in the SI of IND-treated *Sult2b1^−/−^* mice (n = 5 mice per group; two-tailed unpaired Student’s *t*-test). Before IND injection, anti-Ly6G or isotype control antibodies were intraperitoneally administrated **(G)**, and a vehicle or a mixture of ABx was orally administered to the mice **(H)**. Data were obtained from two **(B, F, G)** and three **(C–E, H)** independent experiments, and graphs are shown as the mean ± SD. *P < 0.05; **P < 0.01; ***P < 0.001.

To determine whether CS affects inflammatory status in the SI, we subcutaneously administered a high dose of IND (5 mg/kg body weight) to *Sult2b1^+/+^* and *Sult2b1^−/−^* mice with or without the oral treatment of CS (**Figure 6A**). MS imaging revealed the absence of CS in the crypts around ulcerative lesions of *Sult2b1^+/+^* mice after IND administration (**Figure 6B**). Furthermore, *Sult2b1^−/−^* mice exhibited markedly increased counts and areas of SI ulcers, compared with those in *Sult2b1^+/+^* mice, and these ulcerative lesions were dramatically ameliorated by CS administration (**Figures 6C, D**). Similarly, compared with *Sult2b1^+/+^* mice, the absolute number of neutrophils in ulcerative lesions increased in *Sult2b1^−/−^* mice, which was also suppressed by CS administration (**Figure 6E**). To better understand localization of the administered CS, we orally administered deuterium-labeled CS (d7-CS) to *Sult2b1^+/+^* mice and found that d7-CS was detected in the blood at 1 h after oral administration and localized in the lamina propria and ulcerative lesions lacking endogenous CS 4 h later (**Figure 6F** and **Supplementary Figure 6A**). In addition, when circulating neutrophils were depleted by intraperitoneal injection of anti-Ly6G antibodies (**Supplementary Figures 6B, C**), *Sult2b1^−/−^* mice showed a significant decrease in the counts and areas of SI ulcers (**Figure 6G** and **Supplementary Figure 6D**). Taken together, these results suggest that CS contributes to protecting SI tissues from ulcer formation by suppressing the infiltration of inflammatory neutrophils. In addition, NSAID-induced ulcers are known to be exacerbated by the invasion of commensal bacteria (9, 10). Consistent with this, antibiotic treatment markedly suppressed IND-induced ulcer formation even in *Sult2b1^−/−^* mice (**Figure 6H** and **Supplementary Figure 6E**).

## Discussion

Although the intestinal mucosa is protected from commensal bacteria by several types of barriers, little is known about the mechanism to avoid excessive inflammatory responses in the intestine. In this study, we found that in the SI and colon, CS was mainly produced by IECs and specifically concentrated at the top of the villi close to the intestinal lumen. When *Sult2b1* was genetically deleted, DSS-induced colitis and NSAID-induced SI ulcers were markedly exacerbated, compared with the case of *Sult2b1^+/+^* mice. Thus, CS at the mucosal-luminal interface plays a key role in suppressing inflammatory responses during mucosal injury.

A recent study has reported that IEC-specific *Sult2b1* deletion mice (*Sult2b1* ^f/f^ Villin-Cre mice) develop severe DSS-induced colitis, compared with the control *Sult2b1* ^f/f^ mice (24). Although they suggested that CS alleviates gut inflammation by promoting cholesterol biosynthesis in IECs (24), the effect of CS on intestinal immune cells remains unexplored. DOCK2 is a Rac activator predominantly expressed in hematopoietic cells. We previously demonstrated that CS suppresses migration and activation of immune cells by inhibiting DOCK2-mediated Rac activation (26). Importantly, we found in this study that the degree of weight loss during DSS-induced colitis was comparable between *Sult2b1^+/+^* and *Sult2b1^−/−^* mice when *Dock2* was genetically deleted (**Figure 3**). These results suggest that CS limits excessive gut inflammation, at least in part, by inhibiting DOCK2 functions in immune cells.

Neutrophils are essential components of the innate immune response and fight invading bacteria by secreting anti-microbial substances such as ROS (45). However, the accumulation of activated neutrophils in the intestinal tissue can also inflict serious mucosal damage (43, 45). A growing number of studies using single-cell transcriptomics revealed that neutrophil activity in the inflamed intestine was closely associated with the resistance to therapies such as anti-TNF agents and corticosteroids for IBD (46–48). We found that both DSS-induced colitis and IND-induced SI ulcer formation were exacerbated in *Sult2b1^−/−^* mice with increased prevalence of neutrophils (**Figures 2**, **5**, **6**). At this stage, it is difficult to conclude that the increase in neutrophils is a cause of mucosal injury. However, we have also shown that the depletion of neutrophils in *Sult2b1^−/−^* mice attenuated the disease development in both models (**Figures 5**, **6**). In addition, CS inhibited ROS production by neutrophils (**Figure 5**). Therefore, suppression of neutrophil infiltration and ROS production would be one of the mechanisms of CS to limit gut inflammation, although we could not provide evidence that the CS-DOCK2 pathway acts directly on neutrophils *in vivo* in mice with colitis.

Although neutrophils play a key role in DSS-induced colitis and IND-induced SI ulcer formation, the gastrointestinal tract contains many subsets of immune cells other than neutrophils and they express DOCK2. Therefore, it is unlikely that the impact of CS is limited to neutrophils. Indeed, we found that monocytes and macrophages, which are involved in the promotion and resolution of colitis during mucosal injury (49, 50), significantly increased in the colon tissue following the DSS challenge (**Supplementary Figure 4**). In addition, various T cell subsets orchestrate inflammation in IBD by producing pro-inflammatory cytokines (51, 52). Therefore, further studies will be required to elucidate a complete picture of CS–mediated immune suppression in the gut.

In conclusion, our data suggest that IEC-derived CS limits excessive neutrophil recruitment and gut inflammation during mucosal injury. With further investigation, the SULT2B1–CS–DOCK2 axis could be a novel target to develop effective treatments for IBD and NSAID-induced ulcers.

## Data availability statement

The datasets presented in this study can be found in online repositories. The names of the repository/repositories and accession number(s) can be found below: https://bioconductor.org/packages/TabulaMurisData/.

## Ethics statement

The animal study was reviewed and approved by The Ethics Committee on Animal Experiments at Kyushu University.

## Author contributions

KMo, KK, and YF conceived the project. KMo, KK, SA, and KH performed and analyzed most of the experiments. YS, YI, RM, and TB contributed to the measurement and analysis of cholesterol sulfate using mass spectrometry. KMa assisted with the CyTOF experiments. SM and ST generated mutant mice using CRISPR/Cas9 system. YS, TU, and DS provided advice on experimental design and conceptualization. KMo, KK, and YF interpreted and wrote the manuscript. All authors reviewed and approved the final manuscript. All authors contributed to the article and approved the submitted version.
