# Supplementary material for: Cholesterol sulfate limits neutrophil recruitment and gut inflammation during mucosal injury
Source: Front Immunol. 2023 Mar 17;14:1131146. doi: 10.3389/fimmu.2023.1131146 (PMC10063914; doi:10.3389/fimmu.2023.1131146)
Supplement: Supplementary file 1 [file DataSheet_1.docx]

Supplementary Material

Cholesterol sulfate limits neutrophil recruitment and gut inflammation during mucosal injury

## Supplementary Figures

**
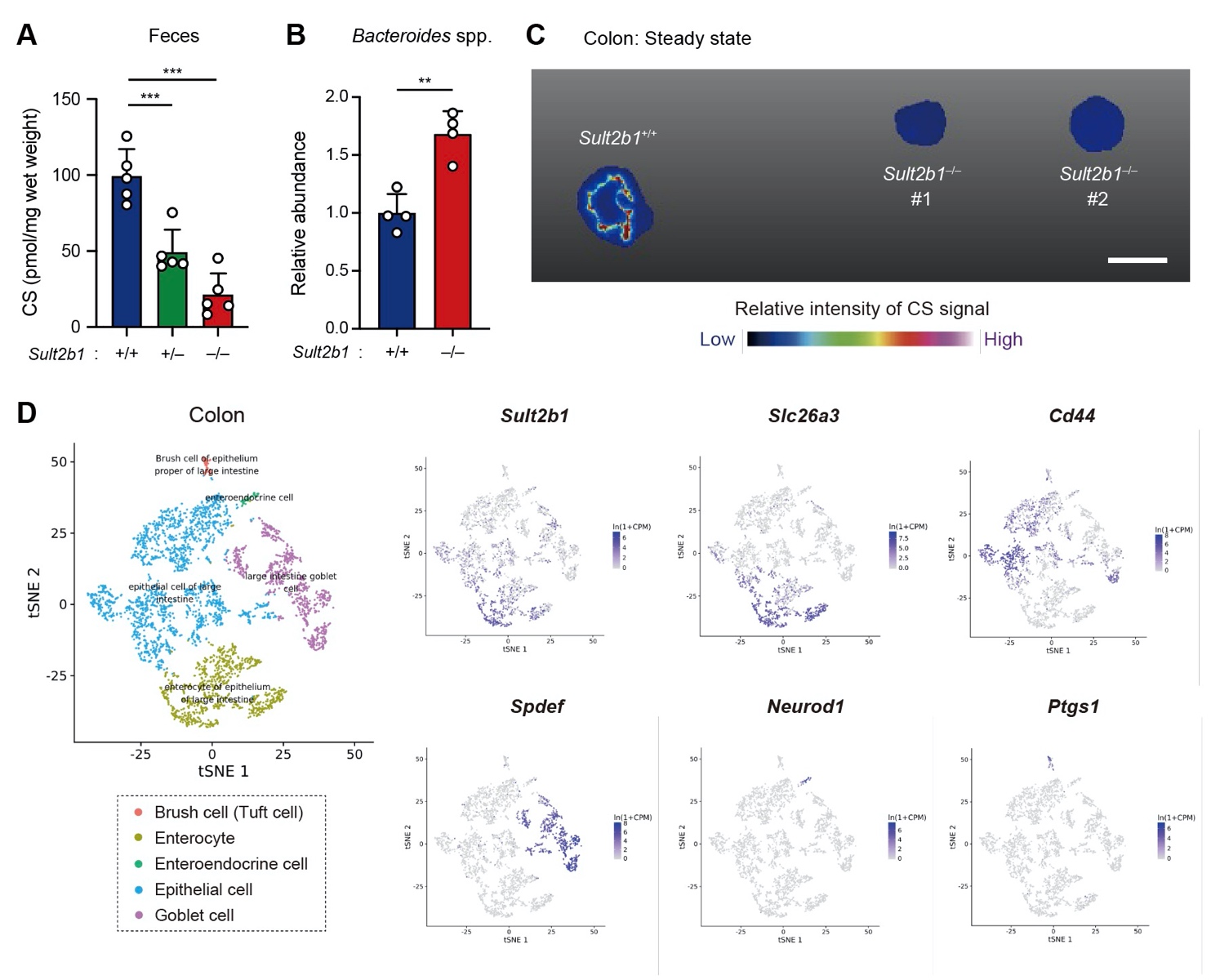
**

**Supplementary Figure 1.** **The detection of cholesterol sulfate (CS) and *Bacteroides* spp. in *Sult2b1^−/−^* mice feces.** **(A)** The CS levels in the feces of *Sult2b1^+/+^*, *Sult2b1^+/−^*, and *Sult2b1^−/−^* mice were quantified using LC-MS/MS (n = 5 mice per group; one-way ANOVA with Dunnett’s multiple comparison test). **(B)** 16S rRNA of *Bacteroides* spp. and total bacteria in feces was quantified using real-time PCR. The abundance of *Bacteroides* spp. relative to that of total bacteria were normalized by setting the mean of relative abundance in *Sult2b1^+/+^* mice as 1 (n = 4 mice per group; two-tailed Mann–Whitney test). **(C)** Mass spectrometry imaging of CS in cross-section of the colon. Samples from *Sult2b1^+/+^* and *Sult2b1^−/−^* mice were placed on the same slide for comparison. Color bar indicates the relative intensities of the CS signal [*m/z* 465]. Scale bar, 3 mm.

**
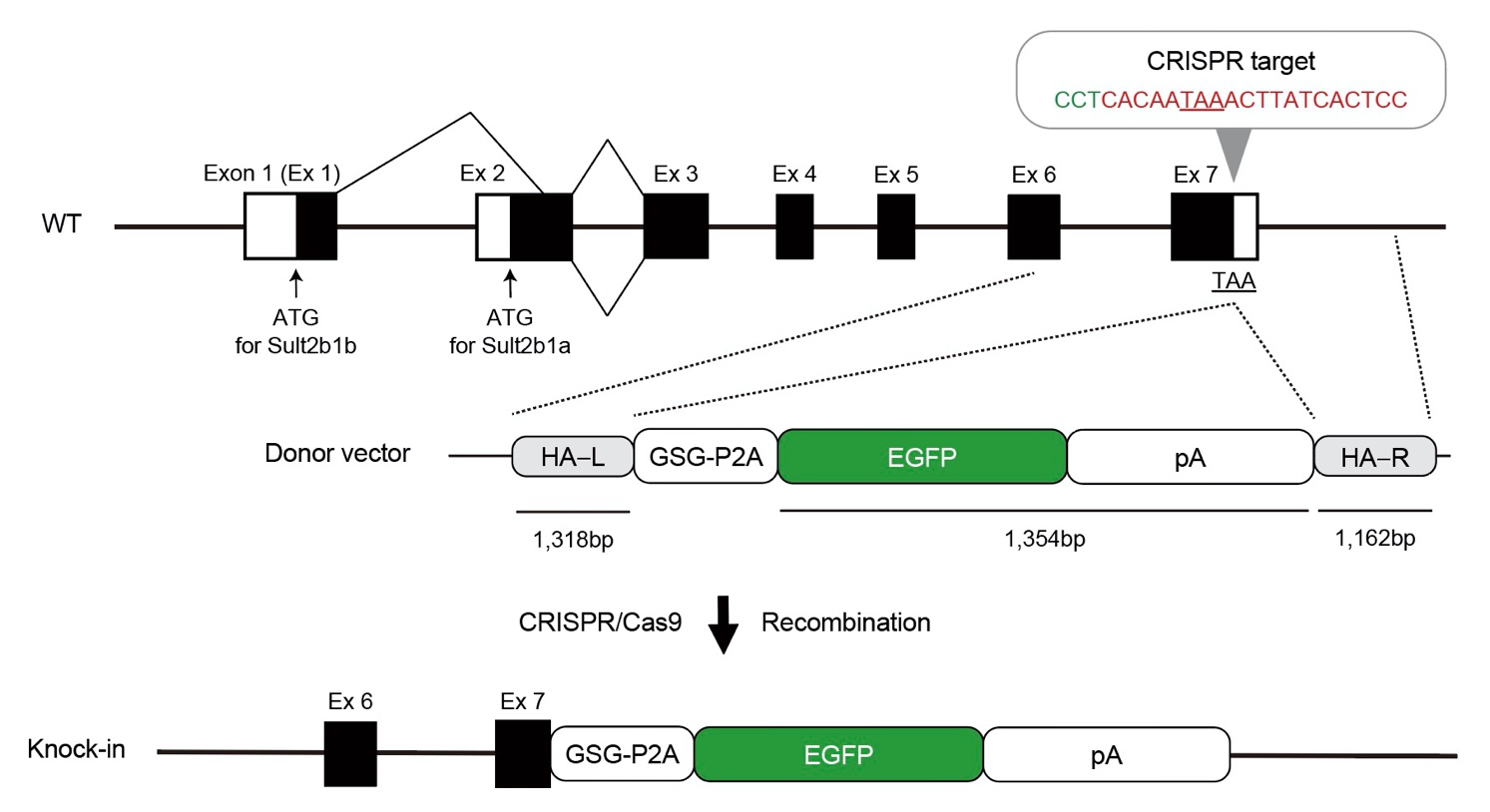
**

**Supplementary Figure 2.** **Schematic illustration of the strategy to insert a P2A-EGFP reporter cassette into the *Sult2b1* locus.** The CRISPR/Cas9 genome editing system was used in combination with gene targeting by homologous recombination. Upper diagram shows the *Sult2b1* wild-type allele, which contains coding sequences (CDS) shown in black boxes and untranslated regions (UTR) shown in white boxes. Red and green letters indicate the single-guide RNA target and protospacer adjacent motif (PAM) sequences, respectively. Central diagram shows the donor vector that contains the left and right homologous arms (HA−L and HA−R), GSG-P2A, EGFP, and rabbit globin polyA (pA). Bottom diagram represents the knock-in allele. Stop codon is shown as underlined letters.

**
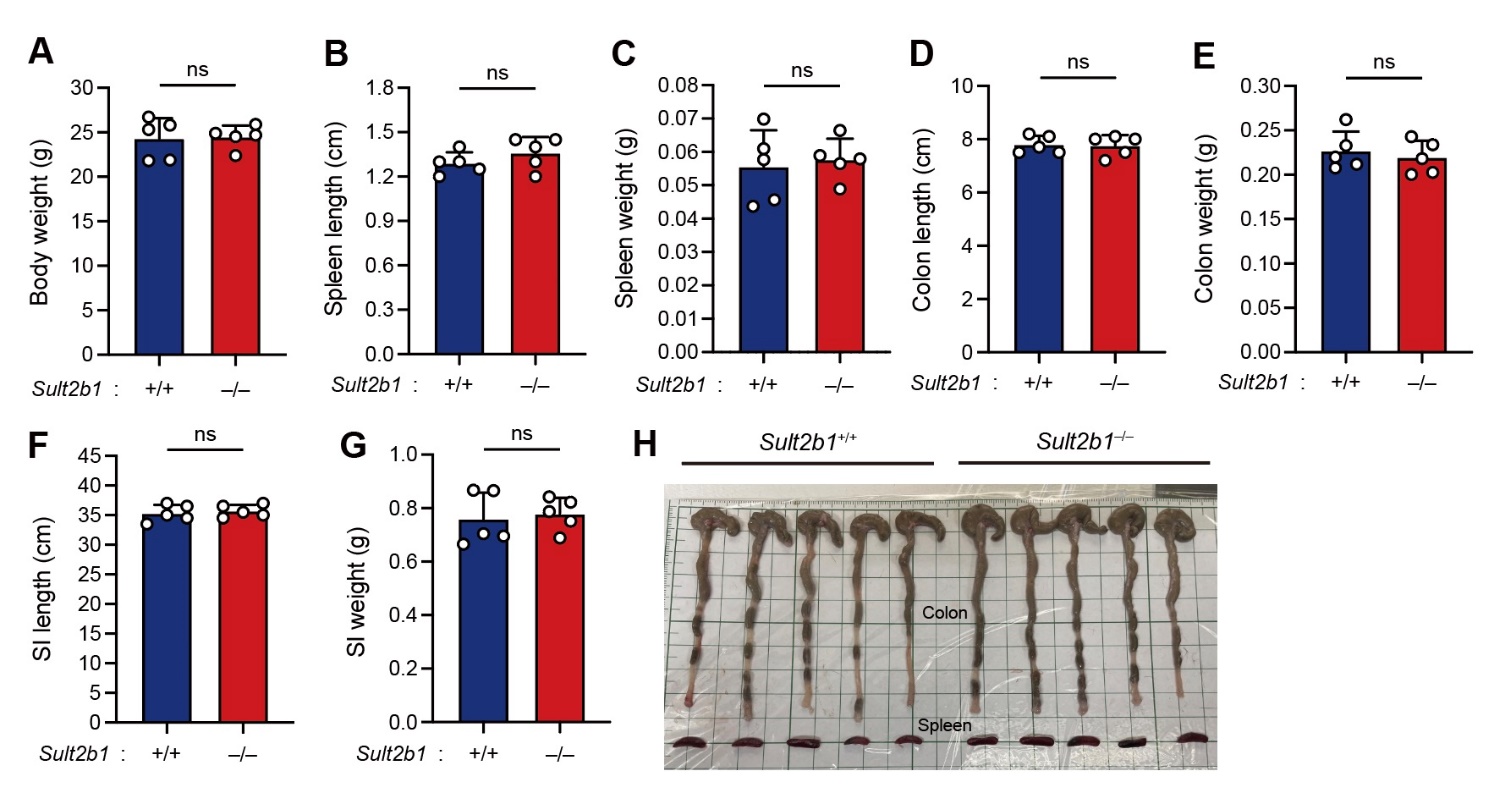
**

**Supplementary Figure 3.** ***Sult2b1^−/−^* mice show no obvious developmental defects compared with *Sult2b1^+/+^* mice.** **(A–G)** The following features were assessed and compared between 9-week-old *Sult2b1^+/+^* and *Sult2b1^−/−^* mice at steady state (n = 5 mice per group; two-tailed unpaired Student’s *t*-test): **(A)** body weight, **(B)** spleen length, **(C)** spleen weight, **(D)** colon length, **(E)** colon weight, **(F)** small intestine (SI) length, and **(G)** SI weight. **(H)** Image showing the comparison of the colon and spleen evaluated in **(B–E)**. Graphs are shown as the mean ± SD. ns, not significant.

**
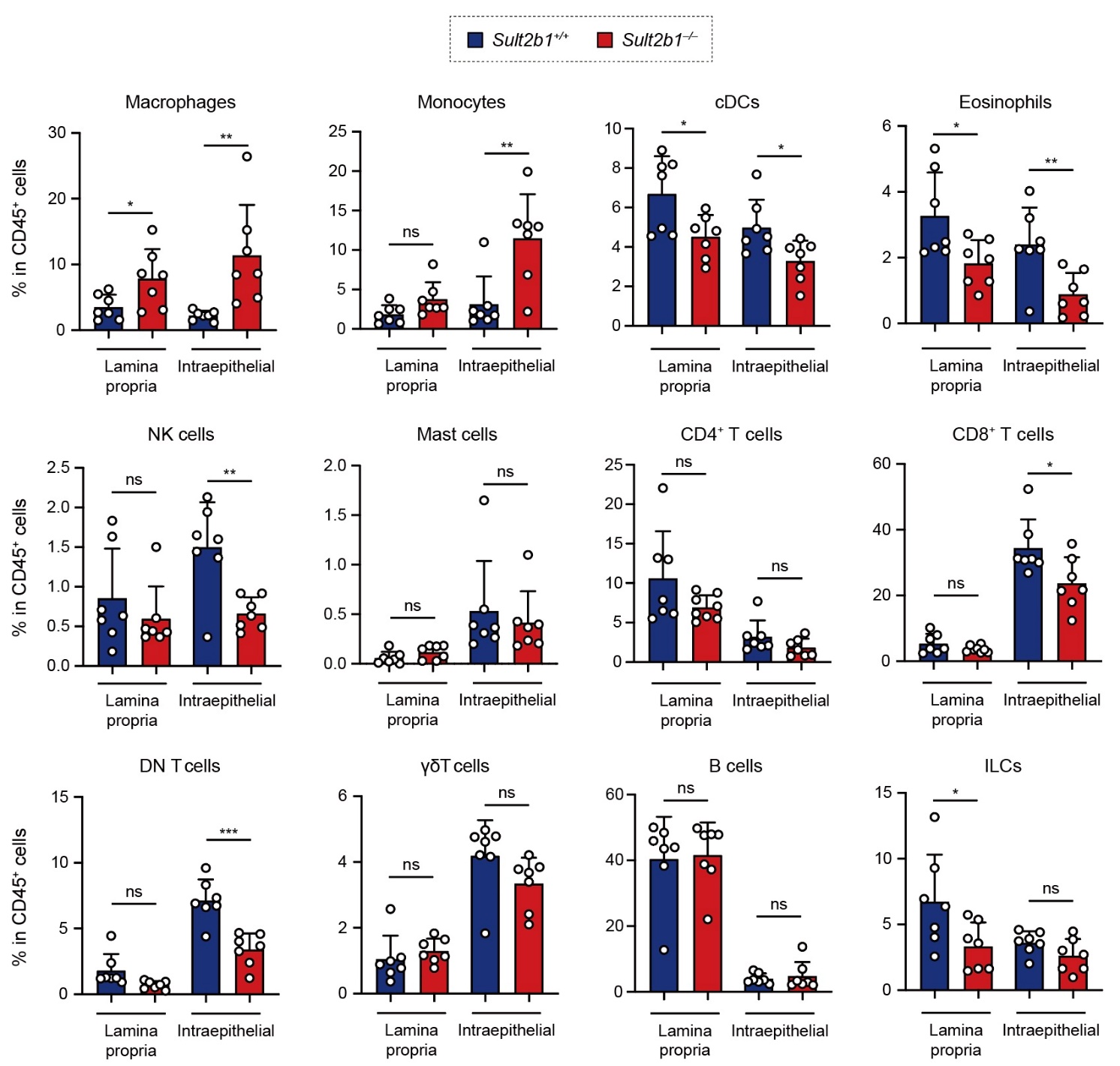
**

**Supplementary Figure 4.** **Percentage of immune cell types to the total intraepithelial and lamina propria CD45^+^ cells.** Each immune cell type was identified using the following markers: B cells (CD45^+^CD3ε^−^CD19^+^MHCII^+^), CD4^+^ T cells (CD45^+^CD3ε^+^CD19^−^CD4^+^), γδT cells (CD45^+^CD3ε^+^

CD19^−^CD4^−^TCRγδ^+^), CD8^+^ T cells (CD45^+^CD3ε^+^CD19^−^CD4^−^TCRγδ^−^CD8α^+^), double-negative T cells (DN T cells; CD45^+^ CD3ε^+^CD19^−^CD4^−^TCRγδ^−^CD8α^−^), eosinophils (CD45^+^CD3ε^−^CD19^−^Gr-1^−^CD11b^+^ Siglec-F^+^), natural killer cells (NK cells; CD45^+^CD3ε^−^CD19^−^Gr-1^−^Siglec-F^−^NK1.1^+^NKp46^+^), mast cells (CD45^+^CD3ε^−^CD19^−^Gr-1^−^Siglec-F^−^NK1.1^−^FcεR1α^+^c-Kit^+^), conventional dendritic cells (cDCs; CD45^+^CD3ε^−^CD19^−^Gr-1^−^Siglec-F^−^NK1.1^−^FcεR1α^−^CD11c^+^

MHCII^high^), monocytes (CD45^+^CD3ε^−^CD19^−^Gr-1^−^Siglec-F^−^NK1.1^−^FcεR1α^−^CD11c^−^CD11b^+^F4/80^−^), macrophages (CD45^+^CD3ε^−^CD19^−^Gr-1^−^Siglec-F^−^NK1.1^−^FcεR1α^−^CD11c^−^CD11b^+^F4/80^+^), and innate lymphoid cells (ILCs; CD45^+^CD3ε^−^CD19^−^Gr-1^−^Siglec-F^−^NK1.1^−^FcεR1α^−^CD11c^−^CD11b^−^

F4/80^−^CD90.2^+^). Data (n = 7 mice per group; two-tailed unpaired Student’s *t*-test) were obtained from three independent experiments, and graphs are shown as the mean ± SD. **P* < 0.05; ***P* < 0.01; ****P* < 0.001; ns, not significant.

**
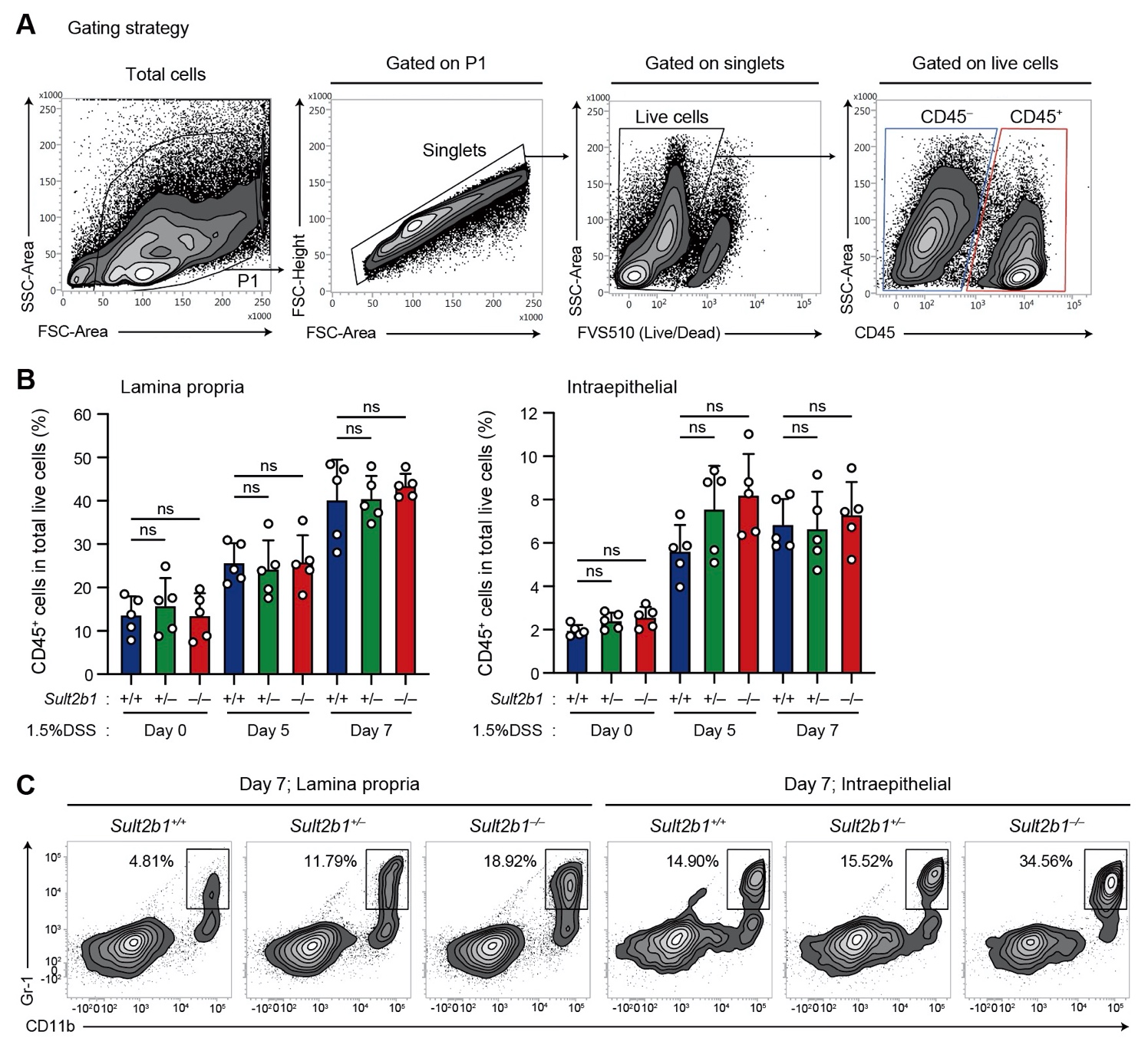
**

**Supplementary Figure 5.** **Gating strategy and percentage of CD45^+^ cells and neutrophils in the colon. (A)** Gating strategy used to identify live CD45^+^ and CD45^−^ cells in the colon. **(B)** Percentage of CD45^+^ cells in total live cells from *Sult2b1^+/+^*, *Sult2b1^+/−^*, and *Sult2b1^−/−^* mice (n = 5 mice per group; one-way ANOVA with Dunnett’s multiple comparison test). **(C)** Flow cytometric analysis of neutrophils in the colon from *Sult2b1^+/+^*, *Sult2b1^+/−^*, and *Sult2b1^−/−^* mice. Numbers indicate the percentage of CD11b^+^ Gr-1^+^ neutrophils in CD45^+^ cells. Data were obtained from five independent experiments **(B, C)**, and graphs are shown as the mean ± SD. ns, not significant.

**
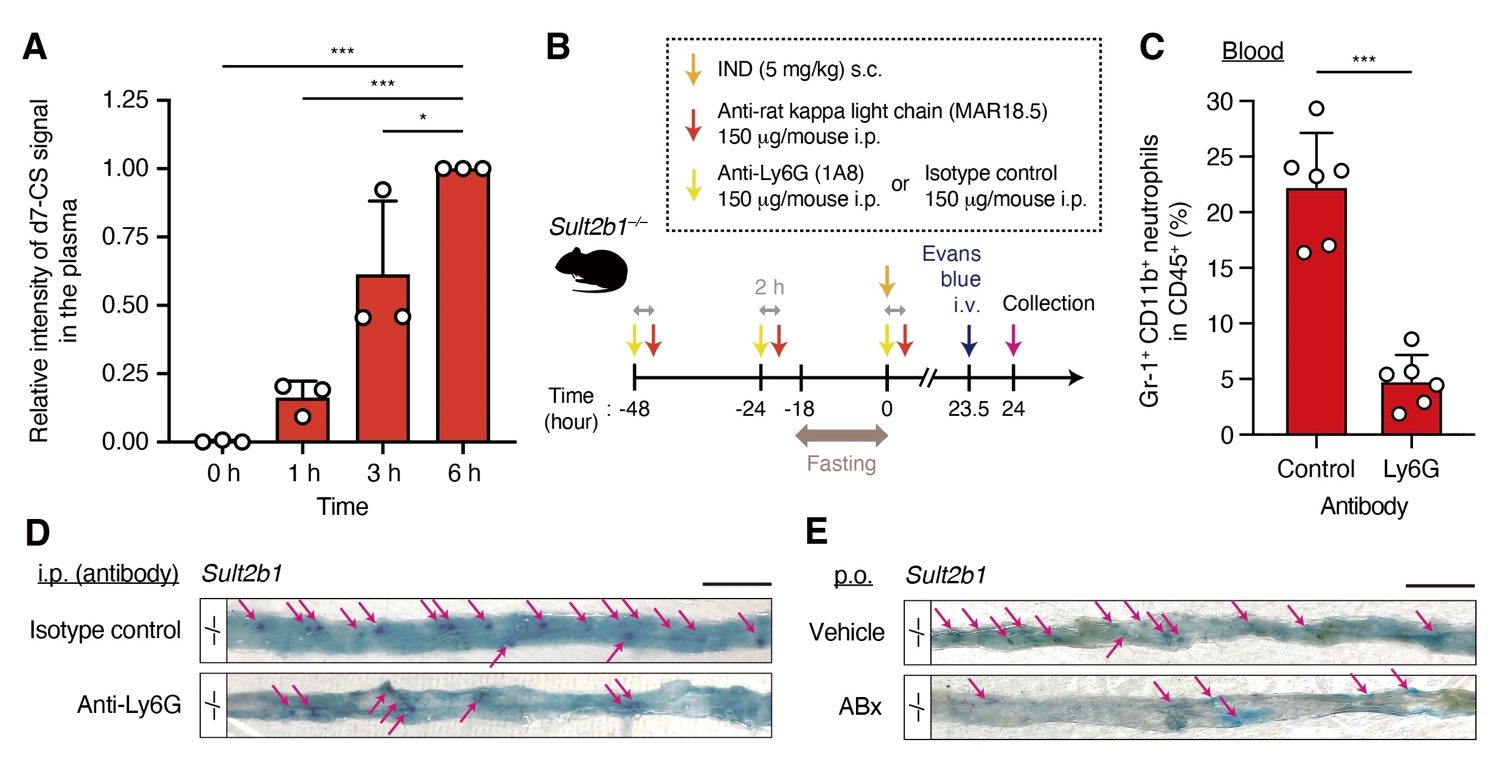
**

**Supplementary Figure 6. Plasma concentration of d7-CS after oral administration and representative images of indomethacin (IND)-induced SI ulcers.** **(A)** The plasma concentration of d7-CS was monitored at 0, 1, 3, and 6 h after d7-CS (330 μg per mouse) was orally administered into wild-type mice. The intensity of the d7-CS signal [*m/z* 472] in plasma at each time point was normalized considering that in plasma at 6 h as 1 (n = 3 mice per group; one-way ANOVA with Dunnett’s multiple comparison test). **(B)** Schematic illustration of the protocol used for circulating neutrophil depletion in the IND-induced SI ulcer model. Indicated two antibodies were intraperitoneally administrated to *Sult2b1^−/−^* mice sequentially at 2-h intervals per day. **(C)** The percentage of CD11b^+^ Gr-1^+^ neutrophils in CD45^+^ cells of the blood from *Sult2b1^−/−^* mice after antibody treatments (n = 6 mice per group; two-tailed unpaired Student’s *t*-test). **(D, E)** Representative macroscopic images of IND-induced SI ulcers in *Sult2b1^−/−^* mice after anti-Ly6G antibodies or antibiotics (ABx) administration. Ulcerative lesions (magenta arrows) are recognized as blue spots in the SI. Scale bar, 10 mm. Data were obtained from one **(A)** and three **(C–E)** independent experiments, and graphs are shown as the mean ± SD. **P* < 0.05; ****P* < 0.001.

**Supplementary Table 1. List of real-time PCR primer sequences.**

| **Gene** | **Forward Sequence (5’- 3’)** | **Reverse Sequence (5’- 3’)** |
| --- | --- | --- |
| *Il6* | ACAAAGCCAGAGTCCTTCAG | TGGAAATTGGGGTAGGAAGG |
| *Il17a* | TAACTCCCTTGGCGCAAAAG | TCTTCATTGCGGTGGAGAGT |
| *Il1b* | GAAGAAGAGCCCATCCTC | GTTCATCTCGGAGCCTGTAG |
| *Tnfa* | TCGTAGCAAACCACCAAGTG | TTTGAGATCCATGCCGTTGG |
| *Tjp1* | ATCAGCACCATGCCTAAAGC | AAAATGCCACGAGCTGTAGC |
| *Ocln* | AGCTTACAGGCAGAACTAGACG | ATTCATCAGCAGCAGCCATG |
| *Muc2* | CCTCGGTCTCCAACATCACC | CCCACAGGACCCAAAACAGT |
| *Lypd8* | ACCATTTTGCAAGCCAGTGC | CGCAGAGTGTTTTGTTGTGACC |
| *Hprt* | CTGGTGAAAAGGACCTCTCG | TGAAGTACTCATTATAGTCAAGGGCA |

| No. | **Metal**  **conjugate** | **Target** | **Amount**  **(μL)** | **Clone** | **Product ID**  **(Fluidigm)** |
| --- | --- | --- | --- | --- | --- |
| 1 | 89Y | CD45 | 1 | 30-F11 | 3089005 |
| 2 | 141Pr | Ly-6G/C (Gr-1) | 1 | RB6-8C5 | 3141005 |
| 3 | 142Nd | CD11c | 1 | N418 | 3142003 |
| 4 | 144Nd | CD45R (B220) | 1 | RA3-6B2 | 3144011 |
| 5 | 145Nd | CD69 | 1 | H1.2F3 | 3145005 |
| 6 | 146Nd | F4/80 | 1 | BM8 | 3146008 |
| 7 | 148Nd | CD11b (Mac-1) | 1 | M1/70 | 3148003 |
| 8 | 149Sm | CD19 | 1 | 6D5 | 3149002 |
| 9 | 151Eu | CD25 (IL-2R) | 1 | 3C7 | 3151007 |
| 10 | 152Sm | CD3e | 1 | 145-2C11 | 3152004 |
| 11 | 153Eu | CD335 (NKp46) | 1 | 29A1.4 | 3153006 |
| 12 | 156Gd | CD90.2/Thy1.2 | 1 | 30-H12 | 3156006 |
| 13 | 159Tb | TCRgd | 1 | GL3 | 3159012 |
| 14 | 163Dy | APC | 1 | APC003 | 3163001 |
| 15 | 165Ho | PE | 1 | PE001 | 3165015 |
| 16 | 168Er | CD8a | 1 | 53-6.7 | 3168003 |
| 17 | 169Tm | Ly-6A/E (Sca-1) | 1 | D7 | 3169015 |
| 18 | 170Er | CD161 (NK1.1) | 1 | PK136 | 3170002 |
| 19 | 172Yb | CD4 | 1 | RM4-5 | 3172003 |
| 20 | 173Yb | CD117 (c-Kit) | 3 | 2B8 | 3173004 |
| 21 | 176Yb | FceRIa | 1 | 1-Mar | 3176006 |
| 22 | 209Bi | I-A/I-E (MHC class II) | 1 | M5/114.15.2 | 3209006 |
|  | **Fluorophore conjugate** | **Target** | **Amount**  **(μL)** | **Clone** | **Product ID** |
| 1 | APC | BST2 (CD317) | 1 | 927 | 127015 |
| 2 | PE | Siglec-F | 1 | E50-2440 | 552126 |

**Supplementary Table 2. CyTOF mass cytometry antibody panel used to examine colonic immune cells.**
